# Biomolecular analysis of the Epigravettian human remains from Riparo Tagliente in northern Italy

**DOI:** 10.1038/s42003-024-06979-9

**Published:** 2024-10-30

**Authors:** Orhan Efe Yavuz, Gregorio Oxilia, Sara Silvestrini, Laura Tassoni, Ella Reiter, Dorothée G. Drucker, Sahra Talamo, Federica Fontana, Stefano Benazzi, Cosimo Posth

**Affiliations:** 1 Archaeo- and Palaeogenetics, Institute for Archaeological Sciences, Department of Geosciences, University of Tübingen, Tübingen, Germany; 2 Senckenberg Centre for Human Evolution and Palaeoenvironment at the University of Tübingen, Tübingen, Germany; 3 Department of Translational Medicine for Romagna, University of Ferrara, Ferrara, Italy; 4 Department of Cultural Heritage, University of Bologna, Ravenna, Italy; 5 Department of Chemistry G. Ciamician, Alma Mater Studiorum, University of Bologna, Bologna, Italy; 6 Biogeology, Department of Geosciences, University of Tübingen, Tübingen, Germany; 7 Dipartimento di Studi Umanistici – Sezione di Scienze Preistoriche e Antropologiche, University of Ferrara, Ferrara, Italy; 8 Department of Archaeogenetics, Max Planck Institute for Evolutionary Anthropology, Leipzig, Germany

**Keywords:** Genetic variation, Computational biology and bioinformatics, Biological techniques

## Abstract

The Epigravettian human remains from Riparo Tagliente in northern Italy represent some of the earliest evidence of human occupation in the southern Alpine slopes after the Last Glacial Maximum. Genomic analyses of the 17,000-year-old Tagliente 2 mandible revealed the oldest presence of a genetic profile with affinities to the Near East in the Italian peninsula, which later became the most widespread hunter-gatherer ancestry across Europe. However, a comparable biomolecular characterization of the Tagliente 1 burial remains unavailable, preventing us from defining its biological relationships with Tagliente 2. Here, we apply paleogenomic, isotopic, and radiocarbon dating analyses on a femur fragment of Tagliente 1 and compare the reconstructed data with previously reported results from Tagliente 2. Despite their different isotopic signatures and non-overlapping radiocarbon dates, we reveal that the two human remains belong to the same male individual. We determine that the distinct isotopic values can be explained by different dietary practices during lifetime, whereas the non-overlapping radiocarbon dates can be caused by minimal radiocarbon contamination, possibly deriving from chemical treatments for conservation purposes. These findings highlight the importance of interdisciplinary biomolecular studies in offering new perspectives on the Palaeolithic fossil record and addressing long-standing bioarchaeological questions.

## Abstract (web summary)

Biomolecular investigation of Epigravettian human remains from Riparo Tagliente in northern Italy using paleogenetics, stable isotopes and radiocarbon dating.

## Introduction

In recent years, research in biomolecular archeology is rapidly expanding, owing to major methodological advancements both at the laboratory and computational levels in the fields of genetics, proteomics, radiocarbon dating and stable isotopes, among others. Despite major progress, biomolecular preservation remains a limiting factor for this type of research. This is likely influenced by many microclimatic factors, but also by time i.e., biomolecular preservation tends to be poorer for older periods^1–3^. Investigations focusing on the European Palaeolithic have found that archeological caves and rock shelters often offer environmental conditions that are favorable for biomolecular preservation, greatly aiding our investigation of early modern human populations^4^. In addition, in those sites where human skeletal remains have been discovered, it is possible to perform biomolecular assays on the retrieved specimens providing direct insights on the date, diet and genomic profile of the analyzed individuals. However, most of these caves and rock shelters were excavated prior to major technological advancements and can now be re-examined with an interdisciplinary approach in an attempt to answer questions that were previously unfeasible to address.

**Fig. 1:**
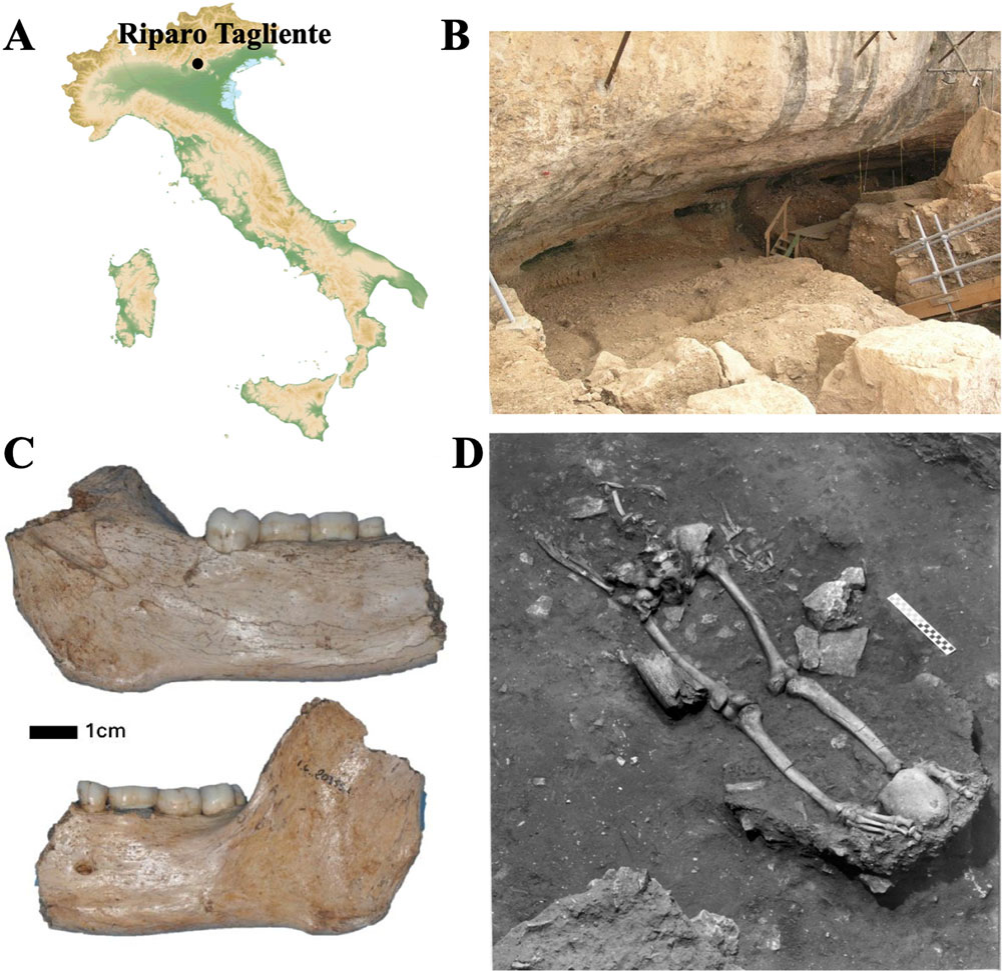
Archeological site and Epigravettian human remains. **A** Topographic map (geospatial data was downloaded using the giscoR package in R) of Italy where the location of the Riparo Tagliente site is indicated with a black dot; (**B**) Photograph of the rock shelter entrance; (**C**) Human hemimandible Tagliente 2; (**D**) Human burial Tagliente 1.

**Fig. 2:**
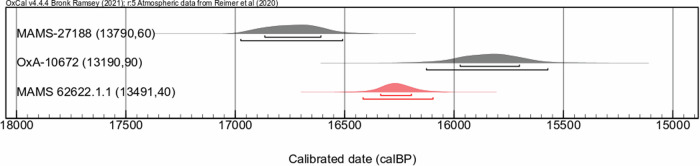
Direct dating. Newly generated radiocarbon date for Tagliente 1 (MAMS 62622.1.1) in red and published radiocarbon dates for Tagliente 2 (MAMS-27188)^5^ and for Tagliente 1 (OxA-10672)^6^ in gray with shorter and longer intervals representing 1σ and 2σ calibration ranges in years before present (cal BP), respectively.

Riparo Tagliente, located in the province of Verona in northeast Italy, is an example of a rock shelter with an abundant Paleolithic zooarchaeological and osteological collection, whose recent re-investigation with radiocarbon dating, isotopic analysis and ancient DNA (aDNA), has proven to be highly informative many years after its initial discovery^5,6^. The rock shelter was first discovered in 1958 by Francesco Tagliente. The site underwent preliminary investigations between 1962 and 1964 under the Museo Civico di Storia Naturale of Verona. In 1967, the University of Ferrara took charge of the excavations and is the main institution to conduct research at the site ever since. The rock shelter’s strategic location at the convergence of diverse topographic formations has yielded extensive knowledge about the interaction between the populations who inhabited the site and their environment, as well as insights into their subsistence strategies^5–13^. The remarkable assemblage of archeological remains found at the site has provided invaluable temporal information, spanning from the Middle Palaeolithic onwards, including the presence of Neanderthal skeletal remains^14^. In the Upper Palaeolithic layers of Riparo Tagliente, other skeletal remains associated with the Late Epigravettian were found. In 1963, a human mandible was discovered in the disturbed external area of the shelter^15^. Ten years later, an incomplete burial, referred to as Tagliente 1, was unearthed within the rock shelter in the southernmost sector of the excavated area (Fig. 1). Bartolomei et al.^7^, conducted a comprehensive descriptive analysis of the Tagliente 1 burial, only consisting of postcranial skeletal elements positioned in a supine orientation and partially covered by calcareous limestone blocks. Morphologically, both findings (the initially discovered mandible and Tagliente 1) have been identified as males with an estimated age at death ranging between 22 and 30 years. However, due to the different stratigraphic locations and the fragmentary nature of the remains, previous investigations attributed the mandible to a distinct individual, referred to as Tagliente 2^15^. This assignment to two individuals is consistent with existing chronological analyses. A rib of Tagliente 1 and a molar of Tagliente 2 have been radiocarbon dated to 16,130–15,570 cal BP (2σ) and 16,980–16,500 cal BP (2σ), respectively^5,6^ indicating a time separation between the two individuals of 890 years using the medians of both calibrated dates (Fig. 2).

Recent aDNA analysis on Tagliente 2 has revealed that this individual carried a genetic profile related to the “Villabruna cluster”, an ancestry that was widespread in Epigravettian-associated individuals from the Italian peninsula and that largely contributed to the vast majority of European populations after ~14,000 years ago^5,16,17^. The presence of this genetic profile in the Alpine region soon after the end of the Last Glacial Maximum (LGM) indicates that it reached Italy thousands of years before spreading into central Europe. In addition to the aDNA work, isotopic analysis conducted on teeth from the Tagliente 2 mandible showed that the individual had a very high protein diet, similar to what is observed for contemporaneous individuals^13^. For Tagliente 1, apart from the younger radiocarbon date, isotopic values pointed to a substantial amount of aquatic protein sources in this individual’s diet^6^. A paleogenetic study has, however, never been conducted on this specimen. In this study, our objective is to align the biomolecular information obtained from Tagliente 1 with that of Tagliente 2. We applied state-of-the-art protocols on a femur fragment of Tagliente 1 to obtain aDNA data from this specimen, alongside newly generated isotopic and radiocarbon dating results. We then compared the genomic, temporal, and stable isotopic profiles of Tagliente 1 and Tagliente 2, to clarify the relationships between these two Late Epigravettian human remains within an interdisciplinary framework.

## Results

**Fig. 3:**
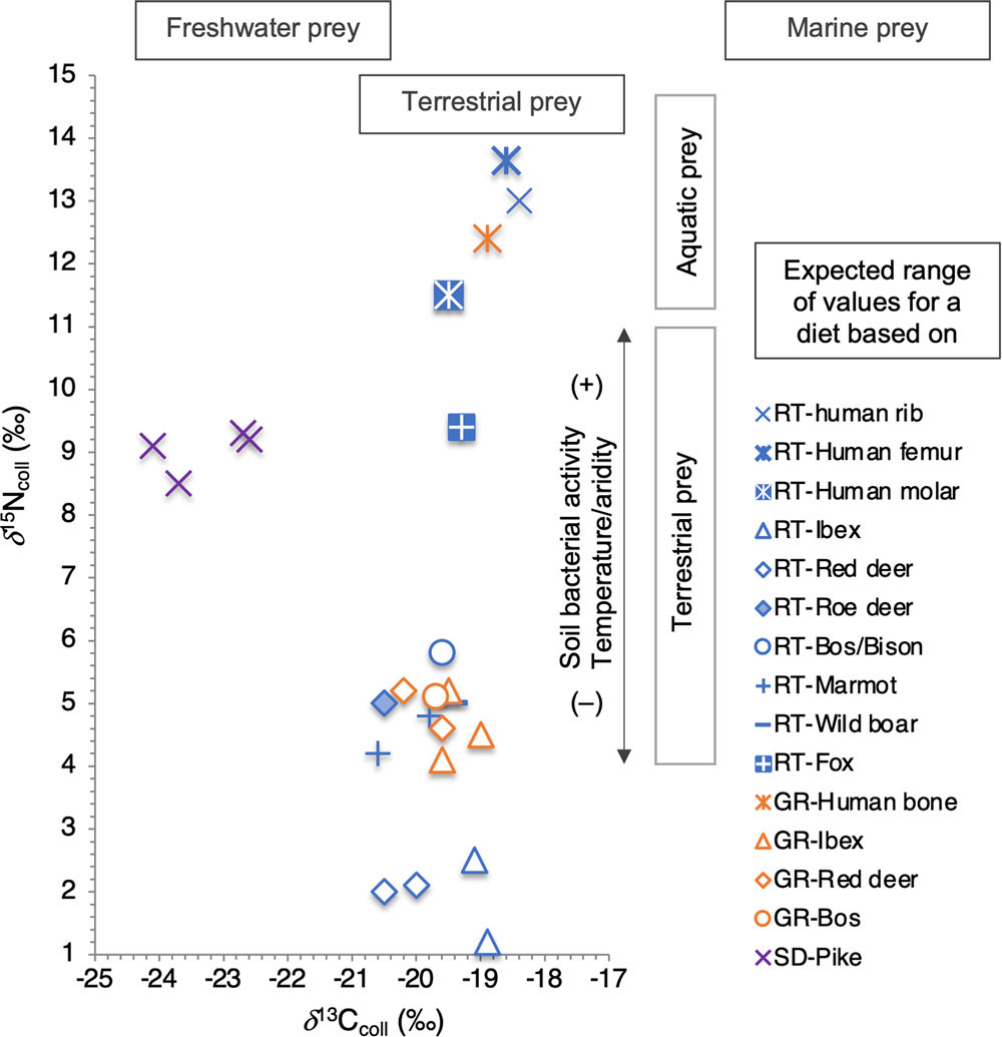
Stable isotopes. Isotopic values of carbon and nitrogen isotopes (δ^13^C and δ^15^N) from the collagen of Epigravettian human and/or animal remains from Riparo Tagliente (RT) in northern Italy, Grotta del Romito (GR) in southern Italy, and Šandlja II (SD) in Croatia.

The new AMS date on the femur of Tagliente 1 yielded an age of 16,360-16,210 cal BP at 68.3% and 16,460-16,130 cal BP at 95.4% probability (Supplementary Data 1 and Fig. 2). This date has a median value which is ~450 years older than the previous date on a rib of Tagliente 1^6^. However, the newly generated date is still ~450 years younger than what obtained for a first molar of Tagliente 2 and not overlapping at 2σ probability^5^. We then investigated the possibility that this temporal difference could be influenced by different dietary practices during the individual’s lifespan. In fact, the dentine of the first molar does not remodel after its completed growth, between the third and fifth year of life, and it retains an early dietary signal that may be impacted by milk consumption before complete weaning. It can thus be suspected that the dentine of the first molar has an elevated *δ*^13^C and *δ*^15^N value compared to continuously remodeling bones. In contrast, ribs are among the bones with the fastest collagen turnover, meaning that they would reflect the last years of the dietary life of the individual. Femurs show a slower turnover and thus isotopically record more than the last ten years of the individual’s diet^18^. We then performed a new stable isotope measurement on the collagen extracted from the femur of Tagliente 1. The *δ*^13^C and *δ*^15^N values (–18.5‰, +13.5‰, respectively) are consistent with what previously obtained from a rib of the same burial (*δ*^13^C = –18.4‰, *δ*^15^N = + 13.0‰)^6^ (Fig. 3, Supplementary Data 2). When compared with the stable isotopic measurements obtained from the fauna in Riparo Tagliente^6^, the results from both Tagliente 1 bones confirm the higher than expected positive shift of ca. 1‰ *δ*^13^C and 3 to 5‰ *δ*^15^N between the collagen of the consumer and those of its supposed prey^19^. This was interpreted as Tagliente 1 having consumed aquatic resources^6^. Importantly, an aquatic based diet could lead to a reservoir effect on the radiocarbon dating resulting in artificially older dates^20^. However, since the isotopic values from the first molar of Tagliente 2 (*δ*^13^C = −19.5‰, *δ*^15^N = 11.5‰)^13^ are lower than those obtained from the bone fragments of Tagliente 1 (rib and femur), a possible reservoir effect on the radiocarbon date of Tagliente 2 should also be lower. Taken together, the fact that the newly generated radiocarbon date of Tagliente 1 is significantly younger than Tagliente 2 cannot be explained by a higher fresh-water reservoir effect, which would instead make the specimen’s date artificially older.

To assess if the Epigravettian-associated mandible and postcranial remains from Riparo Tagliente are biologically related, we generated genomic data from the femur of Tagliente 1. The initial shallow shotgun screening of the genomic library yielded ~4.5 M raw reads, of which 0.51% were mapped to the human reference genome (hg19) after quality filtering. The average fragment length of the mapped reads was ~58 bp with a CtoT substitution rate of 8.1% at the 5’ end of partially UDG-treated DNA, suggesting the presence of aDNA in this sample. We then used an in-solution capture approach to enrich both the complete mitochondrial DNA and ~1.24 M SNPs across the human genome (mtDNA and 1240K captures, respectively). The targeted enrichment produced an average mtDNA coverage of 5.3X and a nuclear DNA coverage on the 1240K capture panel of 0.14X, resulting in 150,334 SNPs. We determined the individual to be genetically male and estimated 6 ± 4% and 26.9 ± 7.3% mtDNA and nuclear DNA contamination, respectively. After utilizing PMDtools to reduce contamination by filtering for deaminated reads, we retained a total of 6317 SNPs (see Methods section).

We determined the mtDNA haplogroup of Tagliente 1 to U2′3′4’7’8’9, which is the same haplogroup obtained from Tagliente 2^5^. This mtDNA haplogroup is commonly observed in the Palaeolithic European record, with particularly high frequency in the Italian peninsula^16,21–24^. We then assigned the Y-chromosome haplogroup to I2, which is also found in Tagliente 2^5^. This Y-chromosome haplogroup rose in frequency during the Upper Palaeolithic to become the dominant paternal marker in younger Epigravettian and Mesolithic Europeans^16,17^.

**Fig. 4:**
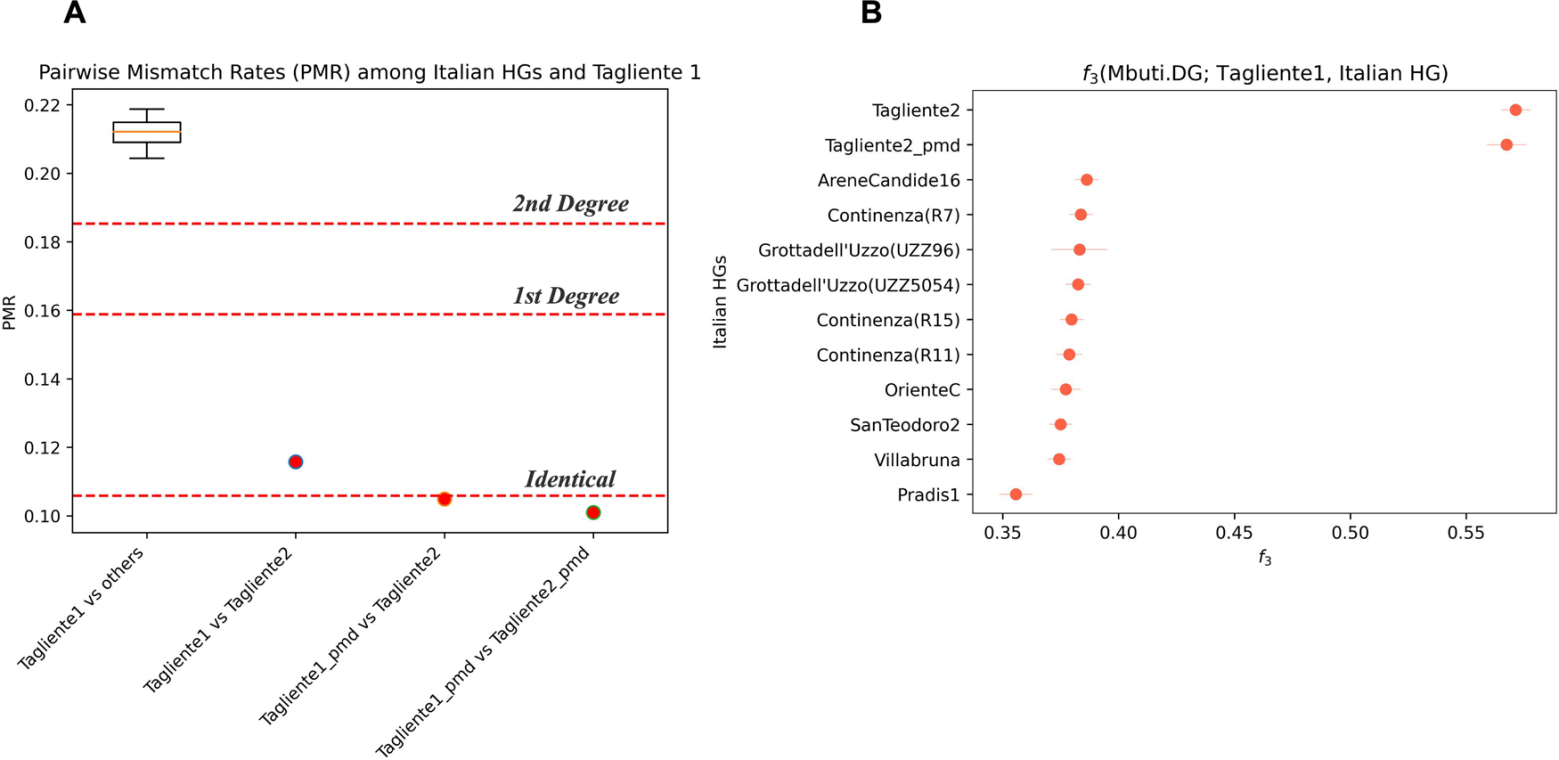
Genetic relatedness and affinity. **A** Pairwise Mismatch Rate (PMR) between Tagliente 1 and other Italian hunter-gatherers carrying “Villabruna” ancestry or Tagliente 2 only. In the box plot the orange line describes the PMR median and box limits the upper and lower quartiles (*n* = 10). **B** Pairwise *f*_*3*_-outgroup statistics between Tagliente 1 and other Italian hunter-gatherers associated with the “Villabruna” ancestry (Mbuti used as outgroup). Error bars represent one standard error.

Subsequently, we calculated the pairwise mismatch rate (PMR) by comparing the genotype of Tagliente 1 to Tagliente 2 and ten other Epigravettian and Mesolithic individuals from Italy belonging to the “Villabruna cluster” (Fig. 4A and Supplementary Data 3). The PMR between Tagliente 1 and the other Villabruna-related individuals (excluding Tagliente 2) has an average value of 0.212. In contrast, the PMR between the two Tagliente remains is determined to be 0.116. Notably, when PMR is calculated between Tagliente 2 and the genotype of Tagliente 1 after PMD filtering or between both Tagliente samples after PMD filtering, the resulting values approach even closer to half of the PMR baseline for unrelated individuals (Fig. 4A and Supplementary Data 3). Since the genotype for each individual is inferred in pseudo-haploid form with equal probability of selecting one allele for each heterozygous SNP, the expected PMR value for identical or twin individuals is half of the PMR baseline^25^. Therefore, based on this analysis, we conclude that Tagliente 1 and Tagliente 2 are either twin individuals or different skeletal elements originating from the same individual.

To confirm this conclusion via a method that provides statistical uncertainty, we calculated *f*_*3*_-outgroup statistics in the form *f*_*3*_(Mbuti; X1, X2), where X1 and X2 are Epigravettian and Mesolithic individuals from Italy carrying “Villabruna” ancestry (Fig. 4B and Supplementary Data 4). When Tagliente 2 is compared to our newly generated data from Tagliente 1, we obtain a *f*_*3*_ value of 0.571 ± 0.006. This value increased (0.619 ± 0.024) when we used the PMD-filtered data from Tagliente 1 (Supplementary Data 4). The resulting shared drift between Tagliente 1 and Tagliente 2, being much higher than the baseline among hunter-gatherer individuals associated with the “Villabruna” ancestry (0.378 on average) (Fig. 4B), can be explained by these two skeletal elements indeed belonging to identical twins or to the same individual. In addition, the fact that the *f*_*3*_-outgroup value between the two Tagliente samples is substantially higher than 0.5 suggests that the population these samples derive genetic ancestry from had low heterozygosity. This likely resulted from a small effective population size, as previously reported for other Epigravettian-associated individuals from Italy^16^.

## Discussion

Inferences using nuclear DNA (sex determination, PMR, *f*-statistics) and uniparental markers (mtDNA and Y-chromosome) all converge on the conclusion that the skeletal remains of Tagliente 1 and Tagliente 2 either belong to identical twins or to the same individual. To distinguish between these two scenarios, we re-evaluate the morphological analyses previously performed on both human remains.

The determination of Tagliente 1 age at death was achieved through the examination of the full fusion of the epiphysis in long bones, indicative of an adult status, as well as an assessment of the pelvic region. The analysis of symphyseal facets revealed the persistence of transverse grooves and distinct characteristics in the anterior and posterior edges of the symphyseal regions^26^. The presence of horizontal crests within the pelvic region and the absence of clear demarcation between the upper and lower boundaries of these facets suggest that the age at the time of death likely fell within the range of 22–24 years^26^. Regarding Tagliente 2, limited information is available due to the fragmentary nature of the mandible and the scarcity of diagnostic morphological features. However, it is plausible to attribute this individual to an age above 25 years but below 30 years, as evidenced by the eruption of the third molar and the limited dental wear. Through morphological analyses, both samples have been identified as males. The strong indication of male sex for Tagliente 1 is inferred from the proportions and shape of the sacrum, which is further supported by the overall morphology of the pelvis. The pelvis exhibits a relatively tall and narrow shape with robust and massive characteristics. Distinctive features include the sharp angulation of the pelvic brim (*linea arcuata*), the triangular appearance of the body of the pubis (*lamina quadrilatera*), and its subpubic angle, which is concave rather than convex downward. The obturator foramen is nearly oval rather than triangular. Additionally, the long bones exhibit robust features, reinforcing the determination to male sex^26^. For Tagliente 2, morphological sex determination is less straightforward due to the absence of several key characteristics typically used for such assessment. However, measurable dimensions, such as the height of the mandibular body and its thickness at the mental foramen level, are more consistent with a male rather than a female attribution^15^. Overall, the newly obtained genomic results, alongside previously performed morphological investigations indicating a male sex and similar age at death, allow us to most parsimoniously conclude that Tagliente 1 and Tagliente 2 belong to one single individual. We thus retract the previous individual names and, to avoid future inaccuracies, we rename the entire fossil as “Tagliente 1/2” (postcranium and mandible).

This result requires explanation of the reasons why the two human remains were for long considered as belonging to two different individuals. The Tagliente burial came from the inner area of the rock shelter, but it was found partially incomplete, with only postcranial elements preserved. In addition, there is evidence that some of the deposits, which had formed in this area during the Late Epigravettian, were displaced before the start of any archeological excavation. This activity was most likely carried out during historical periods in order to create room inside of the shelter, although it is also documented that in the years following the discovery of the site, the Late Epigravettian deposits continued to be dug by local people searching for bones and chert items. The mandible was not found in association with the human burial, but it was accidentally identified within disturbed deposits in the external area of the shelter. In the light of the genomic results presented here, it is likely that the mandible was initially part of the burial, but it was later displaced from the rock shelter through the non-archeological excavation activities mentioned above. Therefore, a probable explanation is that only one individual was buried in the Late Epigravettian deposits and, for this reason, the hypothesis that the postcranium and the mandible belong to the same individual can also be reconciled with the archeological evidence.

Regarding the discrepancy in the isotopic results between the two human remains, it is important to consider the isotopic composition of the faunal baseline. Despite a relatively low number of specimens (*n* = 10), the herbivores from the same site can be separated in two groups based on *δ*^15^N values: one ranging from +4.2 to +5.8‰, values close to the fauna dated to the same period from the Late Epigravettian layers of another Italian site (Grotta del Romito in Fig. 3)^27^, and another one ranging from +1.2 and +2.5‰ (Supplementary Data 2). Since *δ*^15^N values of plants and herbivores decrease with decreasing temperature, aridity, and soil microbial activity, the second group, which includes red deer and ibex in contrast to wild boar and large bovine, can be interpreted as the result of a high elevation habitat^28^. The fox specimen analyzed at Riparo Tagliente shows *δ*^13^C and *δ*^15^N values consistent with the consumption of high *δ*^15^N prey (Fig. 3), interpreted here as low-elevation prey. Interestingly, both human remains, Tagliente 1 and Romito 9, have likely consumed aquatic resources, based on their elevated *δ*^15^N values. Indeed, the archeological pikes from the Istrian site of Šandalja II^29^ illustrate well the typical lower *δ*^13^C and higher *δ*^15^N values of freshwater resources compared with terrestrial resources (Fig. 3). Since the rib of Tagliente 1 and the humerus/femur of Romito 9 did not provide depleted *δ*^13^C values, it was concluded that an additional marine contribution could not be ruled out^6,27^. In all cases, increased consumption of aquatic resources will result in increased *δ*^15^N values, which is not observed in the first molar of the Tagliente 2 mandible^13^ in comparison with both the rib and femur of Tagliente 1. Ribs and limb bones are considered to reflect the dietary signal of the last years of the life of a mature individual as their collagen is continuously remodeled. The difference in *δ*^13^C and *δ*^15^N values between the rib and the femur is indeed less than 0.5‰ and 0.9‰, respectively, as expected from two bones belonging to the same individual^30^. In contrast, the dentine of a first molar, whose sequential formation starts during the first year and is completed until the ninth year of life, will retain an early dietary signal that may be impacted by milk consumption before complete weaning. It is thus expected to observe higher *δ*^13^C and *δ*^15^N values in dentine compared with bone, independently of any other dietary change. Since the contrary is observed here, we can conclude that a higher aquatic consumption is observed in the collagen extracted from the rib and femur than from the molar suggesting a larger reliance on aquatic resources in adulthood rather than in childhood of the Tagliente 1/2 individual.

As mentioned before, an aquatic-based diet would lead to a reservoir effect on radiocarbon dating that could result in artificially older dates. However, a younger date was obtained for the rib and the femur compared to the mandible’s first molar (Fig. 2). Since both postcranial and dental elements belong to the same individual, a distinct aquatic consumption in different phases of life is not a plausible explanation for the difference in radiocarbon ages.

We thus investigated the possibility that ^14^C contamination could be the reason for the inconsistencies in the radiocarbon dates of different skeletal elements of the same individual. Compared to the ^14^C date obtained for the mandible, the previously and newly generated dates on the postcranium are 600 and 280 ^14^C years younger, respectively (Fig. 2). The newly generated date on the femur was produced by following the exact same laboratory procedures as for the dating of the mandible, but two different MICADAS AMS spectrometers were implemented in the dating process. Bartolini et al.^31^, utilized an older generation machine, whereas our new date was obtained with a newer generation AMS spectrometer with enhanced transmission, as well as reduced and more stable blank control values^32–35^. However, despite these two dates being temporally closer compared to the date reported by Gazzoni et al.^6^, they still do not overlap and cannot be statistically combined after a 2-σ calibration (Fig. 2).

Given that the collagen extracted from the mandible’s tooth provided an older radiocarbon date and that ^14^C contamination is more likely to cause an artificially younger date^36^, we consider the date from Bortolini et al.^5^, to be the most reliable one. Thus, assuming the age obtained from the mandible as the true age of Tagliente 1/2, we calculated which levels of modern ^14^C contamination would be necessary to explain such chronological shifts. It was estimated that a modern ^14^C contamination of just 1.4% and 0.7% for the Gazzoni et al.^6^, date and the newly reported date, respectively, would be sufficient to make them artificially younger than the one in Bortolini et al.^5^, (Supplementary Data 1). The most likely source of collagen contamination is post-excavation treatments of the skeletal material for preservation purposes^1^. Further investigations revealed that the restoration work in the 1970s on Tagliente 1 involved the use of acetone for cleaning and the application of glue in areas with fractures (Benedetto Sala, pers. comm.), potentially influencing the dating accuracy. Based on visual inspection, a consolidant treatment of Tagliente 2 is also likely, but the post-excavation taphonomic history of the mandible is less certain and possibly different from Tagliente 1. Nevertheless, the older date obtained for Tagliente 2 implies that even if a consolidant was applied to the mandible for preservation purposes, it might have not infiltrated the tooth root that was used for radiocarbon dating. As far as stable isotopes are concerned, it has been shown that treatments with acetone not exceeding 48 hours do not impact the original collagen *δ*^13^C and *δ*^15^N values^37^. Only glue made with modern collagen may substantially influence carbon and nitrogen stable isotope values. However, to explain a positive shift on both *δ*^13^C and *δ*^15^N values, a marine origin of such a modern consolidant would be necessary (modern terrestrial specimens would provide lower *δ*^13^C values due to the Suess effect), and in a proportion that would much more strongly affect the radiocarbon dating results.

It is concerning that such minimal amounts of ^14^C contamination possibly introduced during restoration procedures could cause three skeletal elements belonging to the same individual to be radiocarbon-dated several generations apart. Given how common consolidation procedures have been performed for skeletal collections deriving from older excavations^38^, it is important to take specific radiocarbon dating results with great caution, especially when the post-excavation taphonomic history of the remains is not known or poorly recorded. Here, we provide an example of how assessing biological relationships through direct paleogenetic investigations of multiple isolated skeletal remains from the same archeological site is a powerful way to provide a reliable temporal constraint.

In summary, we re-investigated the Late Epigravettian human remains from Riparo Tagliente in Northern Italy. We examined the relationship between the Tagliente 1 human burial and the Tagliente 2 isolated mandible using an interdisciplinary approach. Integrating newly generated data through paleogenetics, radiocarbon dating, and stable isotope analyses with previously published archeological and paleoanthropological results, we conclude that Tagliente 1 and Tagliente 2 belong to the same human fossil that was renamed here “Tagliente 1/2”. We further provide compelling explanations to reconcile the apparent discrepancies that exist between the multiple lines of evidence. The separate location where the mandible was recovered can be explained by disturbance at the rock shelter during historical times and provide support for the conclusion that the mandible was initially part of a single human burial. Instead, the distinct stable isotopic signatures likely derived from different dietary practices during the lifetime of the individual while the non-overlapping radiocarbon dates might have been caused by low amounts of modern ^14^C contamination due to the use of consolidants. We conclude that it is crucial to apply paleogenetic techniques alongside other biomolecular disciplines in future bioarchaeological investigations of old osteological collections to provide novel insights into the existing Paleolithic fossil record.

## Methods

### Sampling

Biomolecular analyses of Tagliente 1 were performed after collecting an osteological fragment from the posterior distal region of the right femur. The specimen is curated at the Natural Science Museum in Verona and sampling permits were provided by the Soprintendenza Archeologia Belle Arti e Paesaggio for the Provinces of Verona, Rovigo, and Vicenza (prot. 0016605 23/06/2021, MIC_SABAP-VR_016). The procedure involved creating a space around the specimen to minimize the introduction of external contamination during the sampling procedures. After a thorough tomographic examination to precisely locate the targeted femur area, we proceeded with sampling. The operation started by marking the bone piece, cutting the cortex with a 20 mm diameter and 0.35 mm thickness diamond disc mounted on a red ring contra-angle handpiece, and a micro-motor set at 18 V (150,000 rpm). Once an area of ~2 × 0.5 cm was marked, the grooves were deepened using a 25 mm long and 0.5 mm diameter conical slotted diamond bur. The cuts were then finalized using a No. 12 surgical scalpel and a Buck 5/6 periodontal scalpel, detaching the bone piece completely. This controlled abrasion process allowed for the extraction of a bone fragment of ~1 g.

### Radiocarbon dating

A sub-sample of around 500 mg was pretreated following the method described in Talamo et al.^1^. The sample underwent an ‘acid-base-acid’ (ABA) sequence designed to achieve decalcification, decontamination, and gelatinization of the bone chunks. This sequence was initiated with an initial step using hydrochloric acid (HCl 0.5 M), which was employed to dissolve mineral substances and certain organic impurities.

Once the CO_2_ effervescence ceased, the demineralized sample was rinsed once with ultrapure Milli-Q water. Subsequently, it was subjected to treatment with NaOH 0.1 M for 30 minutes at room temperature. Afterwards, the NaOH was replaced with ultrapure water for another rinsing step. Finally, the water in glass tubes was replaced with HCl 0.5 M, effectively re-acidifying the bone for an additional 15 minutes at room temperature.

Using a heater block, the resultant collagen was dissolved, turning into gelatin in acidic water (HCl pH 3) at 70°C for a duration of 20 hours. The gelatin obtained was initially filtered using Ezee-filter™ separators (Elkay Laboratory, UK) to eliminate particles smaller than 80μm. Following this step, ultrafiltration was performed to effectively separate low molecular weight contaminants and degraded proteins (<30 kDa) from the larger molecules (>30 kDa) (Sartorius VivaSpin® Turbo 15). Ezee-filters and ultrafilters were meticulously pre-cleaned to prevent any contamination risks associated with the filter membranes. After the ultrafiltration process, only the fractions with a molecular weight greater than 30 kDa were frozen for 24 hours and subsequently freeze-dried for 48 hours.

The collagen obtained was then subjected to graphitization at the BRAVHO lab^39^ using the Elemental vario ISOTOPE select coupled to the AGE 3 (Automated Graphitization Equipment, IonPlusAG, Switzerland)^34^. The resulting graphite target was sent to the Curt-Engelhorn-Center for Archaeometry in Mannheim, Germany (CEZA) measured on a MICADAS AMS^31^.

For quality control, an aliquot of a background bone sample (with a radiocarbon age exceeding 50,000 years) was subjected to pre-treatment and dating procedures together with the sample. This was done to monitor and account for any contamination that might have occurred during laboratory processes. The data reduction was carried out using the BATS software^34^. In accordance with standard practice, an additional 1‰ was incorporated into the error calculation of the sample. Radiocarbon date (reported as ^14^C years before present, ^14^C BP) was calibrated using OxCal 4.4^40^ with the IntCal20 calibration curve^41^. The calibrated date ranges (calibrated years before present, cal BP) are reported at 1 and 2σ ranges, corresponding to a 68.3 and 95.4% probability level respectively. Uncalibrated ^14^C dates are presented with their associated 1σ errors (Supplementary Data 1).

We then estimated the level of potential ^14^C contamination in Tagliente 1. Rather than considering the age of the specimens, the Fraction of Modern Carbon-14 (F^14^C) value was used. F^14^C expresses the ratio of ^14^C to ^12^C in an ancient sample compared to the amount of ^14^C to ^12^C in a modern reference standard. A potential contamination in the sample causes a linear shift in the F^14^C value, which is expressed as a fraction or a percentage. Therefore, the percentage of ^14^C contamination can be calculated based on the deviation of the F^14^C value from the expected sample age^1^.

### Stable isotope measurement

The elemental analyses (C, N) and isotopic measurements (*δ*^13^C, *δ*^15^N) were carried out at the CEZA lab in triplicate on the collagen extracted in the BRAVHO lab. The measurement of C and N content and isotopic composition was conducted using a vario ISOTOPE select elemental analyzer coupled to an Isoprime visION isotope ratio mass spectrometer (Elementar GmbH, Langenselbold). The international standards are a marine carbonate (V-Pee Dee Belemnite) for ^13^C and atmospheric nitrogen (AIR) for ^15^N. Isotopic measurements were calibrated using IAEA-CH-6 (*δ*^13^C:–10.45‰), IAEA-CH-7 (*δ*^13^C:–32.15‰), USGS40 (*δ*^15^N:–4.52‰) and USGS41a (*δ*^15^N: + 47.55‰). Measurement errors were smaller than ±0.1‰ for ^13^C and ±0.2‰ for ^15^N values (1σ) based on triplicate analysis of reference material (sulfanilamide, USGS-43, USGS-40, and USGS-41a). Reliability of the *δ*^13^C and *δ*^15^N values can be established by measuring the chemical composition of collagen, with C:N atomic ratio ranging from 2.9 to 3.6^42^ and percentage of C and N above 8% and 3%, respectively^43^ (Fig. 3 and Supplementary Data 2).

### Paleogenetic data generation and processing

DNA was extracted from 53 mg of bone powder following a protocol developed for ancient DNA^44^ and a genetic library was built from 25 µl of extract following the UDG-half protocol, where Uracil-DNA-Glycosidase partly reduces the damage on the aDNA molecules^45^. Negative and positive controls were taken along the entire workflow. Following indexing PCR and subsequent amplifications, the DNA library was both shotgun sequenced and enriched for the whole mitochondrial DNA (mtDNA) and 1.24 million SNPs (1240K capture) across the human genome^46^ and sequenced on Illumina platforms.

After demultiplexing of the raw sequences allowing for a maximum of one mismatch in each index, the EAGER (v1.92.55) pipeline was followed for data processing^47^. The pipeline incorporates AdapterRemoval (v2.2.0) to remove the adapters from both ends of the reads and fragments with a length below 30 bp^48^. Afterwards, BWA (v0.7.12) was used to align the sequences to the hg19 human reference genome^49^ with parameters *-l 16500* and *-q 30*, as well as CircularMapper to realign mitochondrial sequences to the NC_012920.1 mtDNA reference^47^. After the alignment step, the pipeline utilizes DeDup (v0.12.2) to eliminate duplicate sequences that arise due to PCR amplification^47^. Finally, a preliminary authentication control is performed with mapDamage2.0 that quantifies average post-mortem damage using a Bayesian approach in both molecule termini^50^.

The 1240K capture data was trimmed at each end of the reads by two base pairs to avoid post-mortem DNA damage to introduce errors in our downstream analyses using the *trimBam* function of bamUtils (1.0.15)^51^. Using the trimmed data, we inferred the genetic sex of the individual by normalizing the coverages on the X and Y chromosomes with the autosomal coverage^17^.

We used ANGSD-based nuclear contamination estimate tool^52^ that makes use of the heterozygosity rate on the X-chromosome to quantify the possible human DNA contamination. However, due to low SNP coverage (45 SNPs covered twice on the X-chromosome), the nuclear contamination estimation should be treated with caution^17^. Instead, we used *schmutzi*^53^ with -notusepredC –uselength as parameters to jointly assemble a mtDNA consensus sequence and quantify the contamination in the mtDNA. This tool surveys the consensus endogenous mtDNA sequence for possible modern human mtDNA contaminants using a Eurasian database of 256 mtDNA sequences. We determined the mtDNA haplogroup using Haplogrep2^54^, while Y-chromosome haplogroup was assigned with Yleaf^55^.

Genotyping involved randomly selecting an allele call from high-quality bases (with a Phred-scaled base quality score of at least 30) aligned to 1240K SNP positions that were covered at least once. This so-called pseudo-haploid calling is achieved via the *pileupCaller* tool (https://github.com/stschiff/sequenceTools). Following this, the genotyped data were combined with the relevant ancient populations^5,16,17,24,56,57^.

To ensure the reliability of our conclusions in subsequent analyses, we considered the presence of possible modern-day DNA contamination in our sample. We repeated all analyses on genotype data generated after using PMDtools. This tool specifically retains the deaminated DNA reads with CtoT substitutions towards the 3’ end or GtoA substitutions towards the 5’ end^58^. These filtered reads are regarded as originating from the ancient individual, thus minimizing possible influence of DNA contamination.

In order to quantify the background relatedness among individuals sharing similar ancestries with Tagliente 1, we computed a pairwise PMR calculation^25^ (Fig. 4 and Supplementary Data 3). To obtain a normalized allele-sharing estimate for a pair of samples, we computed *f*_*3*_-outgroup statistics using AdmixTools (version 7.0.1)^59^. For each statistic, we used Mbuti as the outgroup (Fig. 4 and Supplementary Data 4).

### Statistics and reproducibility

Detailed information of the statistical analyses carried out as described in the methods section. All analyses can be reproduced by accessing the associated data linked in the Data Availability statement. Sample sizes were not determined in advance. In Fig. 4A, boxplots extend from the lower to upper quartile values of the PMR data with a line at the median. In Fig. 4B, standard errors for *f*_*3*_-outgroup statistics are calculated using the jackknife method outlined in the AdmixTools paper^59^.

### Reporting summary

Further information on research design is available in the Nature Portfolio Reporting Summary linked to this article.

## Supplementary information

Description of Additional Supplementary File

Supplementary Data

Reporting Summary

## Data Availability

The mtDNA and nuclear DNA sequences of Tagliente 1 are available at the European Nucleotide Archive (ENA) under study accession number PRJEB75368. The source data behind the graphs in the paper can be found in Supplementary Data 1-4.
