# Supplementary material for: Biomolecular analysis of the Epigravettian human remains from Riparo Tagliente in northern Italy
Source: Commun Biol. 2024 Oct 30;7:1415. doi: 10.1038/s42003-024-06979-9 (PMC11526120; doi:10.1038/s42003-024-06979-9)
Supplement: Supplementary file 1 — Description of Additional Supplementary File [file 42003_2024_6979_MOESM1_ESM.pdf]

Description Of Additional Supplementary File

File Name: Supplementary Data 1 - 4

Description: Radiocarbon dating data related to Figure 2; Isotopic data related to Figure 3; Pairwise Mismatch Rate (PMR) data related to Figure 4A; f3-outgroup statistics data related to Figure 4B.
