# Supplementary material for: Biomolecular analysis of the Epigravettian human remains from Riparo Tagliente in northern Italy
Source: Commun Biol. 2024 Oct 30;7:1415. doi: 10.1038/s42003-024-06979-9 (PMC11526120; doi:10.1038/s42003-024-06979-9)
Supplement: Supplementary file 3 — Reporting Summary [file 42003_2024_6979_MOESM3_ESM.pdf]

nature portfolio

Corresponding author(s): Orhan Efe Yavuz, Cosimo Posth

Last updated by author(s): 11/09/2024

Reporting Summary

Nature Portfolio wishes to improve the reproducibility of the work that we publish. This form provides structure for consistency and transparency in reporting. For further information on Nature Portfolio policies, see our Editorial Policies and the Editorial Policy Checklist.

Statistics

For all statistical analyses, confirm that the following items are present in the figure legend, table legend, main text, or Methods section.

n/a	Confirmed	
☐	☒	The exact sample size (*n*) for each experimental group/condition, given as a discrete number and unit of measurement
☐	☒	A statement on whether measurements were taken from distinct samples or whether the same sample was measured repeatedly
☐	☒	The statistical test(s) used AND whether they are one- or two-sided<br>*Only common tests should be described solely by name; describe more complex techniques in the Methods section.*
☒	☐	A description of all covariates tested
☐	☒	A description of any assumptions or corrections, such as tests of normality and adjustment for multiple comparisons
☐	☒	A full description of the statistical parameters including central tendency (e.g. means) or other basic estimates (e.g. regression coefficient) AND variation (e.g. standard deviation) or associated estimates of uncertainty (e.g. confidence intervals)
☐	☒	For null hypothesis testing, the test statistic (e.g. *F*, *t*, *r*) with confidence intervals, effect sizes, degrees of freedom and *P* value noted<br>*Give P values as exact values whenever suitable.*
☒	☐	For Bayesian analysis, information on the choice of priors and Markov chain Monte Carlo settings
☒	☐	For hierarchical and complex designs, identification of the appropriate level for tests and full reporting of outcomes
☒	☐	Estimates of effect sizes (e.g. Cohen's *d*, Pearson's *r*), indicating how they were calculated

Our web collection on statistics for biologists contains articles on many of the points above.

Software and code

Policy information about availability of computer code

Data collection	EAGER v.1.92.55<br>BWA v.0.7.12<br>circularmapper v.1.93.5<br>AdapterRemoval v.2.2.0<br>dedup v.0.12.2<br>mapDamage v.2.0.9<br>samtools v.1.3<br>pileupCaller v.1.4.0.2<br>PMDtools v.0.6<br>EIGENSOFT v.7.2.1 (convertf)<br>bamUtils 1.0.15
Data analysis	schmutzi<br>Yleaf<br>Haplogrep2<br>AdmixTools 7.0.1 (qp3Pop)

For manuscripts utilizing custom algorithms or software that are central to the research but not yet described in published literature, software must be made available to editors and reviewers. We strongly encourage code deposition in a community repository (e.g. GitHub). See the Nature Portfolio guidelines for submitting code & software for further information.

Data

Policy information about availability of data

All manuscripts must include a data availability statement. This statement should provide the following information, where applicable:

- Accession codes, unique identifiers, or web links for publicly available datasets
- A description of any restrictions on data availability
- For clinical datasets or third party data, please ensure that the statement adheres to our policy

The mtDNA and nuclear DNA sequences of Tagliente 1 are available at the European Nucleotide Archive (ENA) under study accession number PRJEB75368.

Research involving human participants, their data, or biological material

Policy information about studies with human participants or human data. See also policy information about sex, gender (identity/presentation), and sexual orientation and race, ethnicity and racism.

Reporting on sex and gender	The research did not involve present-day human participants.
Reporting on race, ethnicity, or other socially relevant groupings	The research did not involve present-day human participants.
Population characteristics	The research did not involve present-day human participants.
Recruitment	The research did not involve present-day human participants.
Ethics oversight	The research did not involve present-day human participants.

Note that full information on the approval of the study protocol must also be provided in the manuscript.

Field-specific reporting

Please select the one below that is the best fit for your research. If you are not sure, read the appropriate sections before making your selection.

☒ Life sciences ☐ Behavioural & social sciences ☐ Ecological, evolutionary & environmental sciences

For a reference copy of the document with all sections, see nature.com/documents/nr-reporting-summary-flat.pdf

Life sciences study design

All studies must disclose on these points even when the disclosure is negative.

Sample size	We performed all population genomics analyses on an ancient human individual, starting from the extraction of ancient DNA from a femur fragment. The studied skeletal remain came from Riparo Tagliente in the province of Verona, Italy.
Data exclusions	We did not exclude any data for the downstream analysis. We repeated the genetic analyses on data obtained after filtering for molecules that showed the signature of post-mortem damage.
Replication	Replication is achieved by performing analyses on genome-wide single nucleotide polymorphisms (SNPs) and merging the newly produced ancient DNA data with the 1240K SNP panel.
Randomization	We applied different quality controls including the authentication of ancient DNA (human DNA proportion, ancient DNA damage and contamination estimates). The sample was not allocated into experimental groups but analyzed individually.
Blinding	No blinding was performed. No group allocation was performed during data collection.

Reporting for specific materials, systems and methods

We require information from authors about some types of materials, experimental systems and methods used in many studies. Here, indicate whether each material, system or method listed is relevant to your study. If you are not sure if a list item applies to your research, read the appropriate section before selecting a response.

Materials & experimental systems

n/a	Involved in the study
☒	☐ Antibodies
☒	☐ Eukaryotic cell lines
☐	☒ Palaeontology and archaeology
☒	☐ Animals and other organisms
☒	☐ Clinical data
☒	☐ Dual use research of concern
☒	☐ Plants

Methods

n/a	Involved in the study
☒	☐ ChIP-seq
☒	☐ Flow cytometry
☒	☐ MRI-based neuroimaging

Palaeontology and Archaeology

Specimen provenance

Access to human remains of Tagliente 1 was granted by Italian Ministry of Culture and Soprintendenza Archeologia Belle Arti e Paesaggio for the Provinces of Verona, Rovigo, and Vicenza (prot. 0016605 23/06/2021, MIC_SABAP-VR_016).

Specimen deposition

The femur fragment analyzed in this study as well as DNA extracts and genetic libraries are stored by the Archeo- and Paleogenetics group at the Institute for Archaeological Sciences, Department of Geosciences, University of Tübingen.

Dating methods

We directly radiocarbon-dated the femur fragment also used for ancient DNA and stable isotope analyses. The collagen obtained was subjected to graphitization at the BRAVHO lab using the Elemental vario ISOTOPE select coupled to the AGE 3 (Automated Graphitization Equipment, IonPlusAG, Switzerland). The resulting graphite target was sent to the Curt-Engelhorn-Center for Archaeometry in Mannheim, Germany (CEZA) measured on a MICADAS AMS31. Pretreatment processes, quality control protocols, and dating methods performed are provided in the Methods section.

☒ Tick this box to confirm that the raw and calibrated dates are available in the paper or in Supplementary Information.

Ethics oversight

The Natural Science Museum in Verona and the Soprintendenza Archeologia Belle Arti e Paesaggio for the Provinces of Verona, Rovigo, and Vicenza approved and provided guidance on the study protocol.

Note that full information on the approval of the study protocol must also be provided in the manuscript.

Plants

Seed stocks

Report on the source of all seed stocks or other plant material used. If applicable, state the seed stock centre and catalogue number. If plant specimens were collected from the field, describe the collection location, date and sampling procedures.

Novel plant genotypes

Describe the methods by which all novel plant genotypes were produced. This includes those generated by transgenic approaches, gene editing, chemical/radiation-based mutagenesis and hybridization. For transgenic lines, describe the transformation method, the number of independent lines analyzed and the generation upon which experiments were performed. For gene-edited lines, describe the editor used, the endogenous sequence targeted for editing, the targeting guide RNA sequence (if applicable) and how the editor was applied.

Authentication

Describe any authentication procedures for each seed stock used or novel genotype generated. Describe any experiments used to assess the effect of a mutation and, where applicable, how potential secondary effects (e.g. second site T-DNA insertions, mosiacism, off-target gene editing) were examined.
